# Learning to remember: The early ontogeny of episodic memory

**DOI:** 10.1016/j.dcn.2013.12.006

**Published:** 2014-01-13

**Authors:** Sinéad L. Mullally, Eleanor A. Maguire

**Affiliations:** a Institute of Neuroscience, Faculty of Medical Sciences, Newcastle University, United Kingdom; b Wellcome Trust Centre for Neuroimaging, Institute of Neurology, University College London, United Kingdom

**Keywords:** Memory development, Infantile amnesia, Hippocampus, fMRI, Episodic memory, Navigation

## Abstract

Over the past 60 years the neural correlates of human episodic memory have been the focus of intense neuroscientific scrutiny. By contrast, neuroscience has paid substantially less attention to understanding the emergence of this neurocognitive system. In this review we consider how the study of memory development has evolved. In doing so, we concentrate primarily on the first postnatal year because it is within this time window that the most dramatic shifts in scientific opinion have occurred. Moreover, this time frame includes the critical age (∼9 months) at which human infants purportedly first begin to demonstrate rudimentary hippocampal-dependent memory. We review the evidence for and against this assertion, note the lack of direct neurocognitive data speaking to this issue, and question how demonstrations of exuberant relational learning and memory in infants as young as 3-months old can be accommodated within extant models. Finally, we discuss whether current impasses in the infant memory literature could be leveraged by making greater use of neuroimaging techniques, such as magnetic resonance imaging (MRI), which have been deployed so successfully in adults.

## Highlights

• We review literature on the ontogeny of episodic memory in the first postnatal year.
• We discuss several extant points of contention.
• One of which involves the status of hippocampal function.
• We highlight the potential usefulness of MRI in progressing points of debate.

## 1 Introduction

“You have to begin to lose your memory, if only in bits and pieces to realise that memory is what makes our lives. Life without memory is no life at all…our memory is our coherence, our reason, our feeling, even our action. Without it, we are nothing….” (Bunuel, 1983).

The above quotation attempts to describe life without memory. Similar sentiments have been articulated time and time again by those who have suffered memory loss in adulthood, and studies of amnesic patients have confirmed the devastation that severe memory impairment imposes on people's lives. And yet, spend time with a young infant, who is considered by many to possess the mnemonic capabilities of a severely amnesic patient (Schacter and Moscovitch, 1984), and it is evident that the sentiments expressed above are not apposite (Rovee-Collier, 1997). Infants are not, as William James (1890) proposed, living in a state of ‘blooming, buzzing confusion’. On the contrary, they appear to possess a remarkable capacity to encode and retain knowledge that is appropriate for their current needs (Spear, 1984). For instance, shortly after birth, the human neonate can distinguish its mother's voice (DeCasper and Fifer, 1980) and learn to modify its sucking behaviour in response to milk reinforcement (Sameroff, 1971). By 3- to 4-days-old, it can recognise its mother's face (Bushnell et al., 1989), and by 8- to 10-days-old it can discriminate its mother's breast milk from that of another mother (MacFarlane, 1975). However, in stark contrast to this mnemonic ability, the human adult will almost certainly be unable to recollect a single episode from their infancy, because during this period the human infant (along with many other species) is considered to suffer from a profound form of memory loss known as infantile amnesia (Howe and Courage, 1993).

In this review we examine the main theoretical framework, adapted from the adult literature, that has attempted to account for these apparent disparities. We discuss the successes and failures of this approach, and ask whether impasses that exist today in the infant memory literature could be leveraged by making greater use of neuroimaging techniques, such as magnetic resonance imaging (MRI), that have been deployed so successfully in adults. We have much to gain by elucidating memory in infancy and early childhood. Knowing what the very young are capable of encoding and retaining over different time periods can inform the educational needs of these populations. In so doing it can guide public policy, for example, by highlighting the benefits that early stimulation, enriched environments and varied experience have on the flexibility and development of infant memory (Cuevas et al., 2006). There are also implications for how young children are dealt with by the legal system, such as the impact of cross-examination on children's testimony (Zajac and Hayne, 2003, Hayne, 2007a). Understanding the maturation of the memory system over time, the interactions between it and the emergence of other cognitive processes, such as episodic future thinking and spatial navigation, could also enhance our understanding of these processes in the adult brain.

## 2 Early theoretical influences

**Fig. 1:**
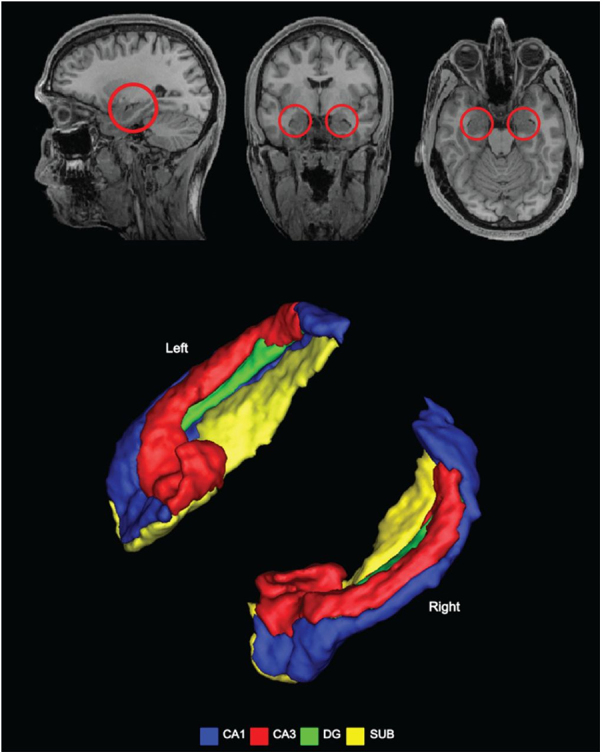
The human hippocampus. The top panel shows the hippocampi circled in red on sagittal (left), coronal (middle) and axial (right) views from a structural MRI brain scan. The hippocampus is composed of a number of subfields, CA1, CA2, CA3, which are adjoined by neighbouring areas – the dentate gyrus (DG), the subiculum (SUB), presubiculum, parasubiculum, and entorhinal cortex – to form the extended hippocampal formation. Three-dimensional images of two example hippocampi are shown in the bottom panel with some of the subregions indicated. From Mullally and Maguire (2013). (For interpretation of the references to color in this figure legend, the reader is referred to the web version of the article.)

The major theoretical influences in the infant memory literature, as it stands today, can be traced back to 1984 when a number of influential papers on infant memory were published. Two of these papers (Nadel and Zola-Morgan, 1984, Schacter and Moscovitch, 1984; see also Bachevalier and Mishkin, 1982) are directly tied to the radical shift in the conceptualisation of memory that was occurring in the adult literature at this time, namely the move away from considering memory as a unitary entity (Squire, 2004). The roots of this departure are grounded in the cognitive and memory profile of one patient in particular – patient H.M. (Scoville and Milner, 1957). At age 27 H.M. underwent bilateral resection of the medial temporal lobes (MTL) to treat intractable epilepsy. This rendered him densely amnesic for new experiences (episodic memories). On this basis the MTL, and in particular the hippocampus (Fig. 1), were identified as critical for the successful acquisition and recollection of episodic memories. H.M.’s ability to acquire new procedural skills such as mirror drawing (Milner, 1962) pointed to a multiple systems account of long-term memory. Although this latter implication was not fully appreciated at the time (because motor memory was considered to be a special, less cognitive, form of memory), intact skills in amnesic patients were subsequently documented across a wide range of perceptual and cognitive tasks (Cohen and Squire, 1980).

**Fig. 2:**
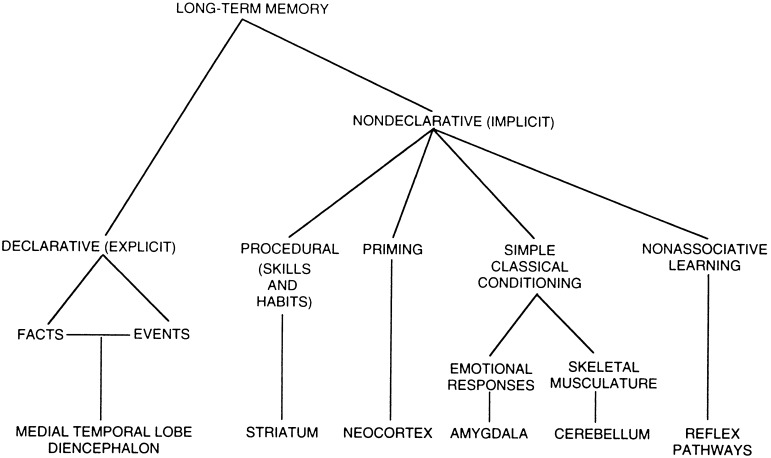
A taxonomy of long-term memory together with the brain structures purported to be involved in supporting each system. As illustrated, declarative memory can be further decomposed into memory for facts (semantic memory) and memory for events (episodic memory). From Squire and Zola-Morgan (1996), © National Academy of Sciences.

These demonstrations, coupled with findings from the animal literature which indicated that the hippocampus supports specific types of memory (e.g. Gaffan, 1974, Hirsh, 1974, O’Keefe and Nadel, 1978, Squire and Zola-Morgan, 1983), led to the idea that there were multiple memory systems (Tulving, 1985). These were subsequently assimilated into a biological framework that listed the memory type along with the supporting brain structures (Fig. 2). In essence, this taxonomy grouped all memory systems that appeared to be preserved in amnesia (and in animals with hippocampal lesions) under the umbrella term ‘nondeclarative memory’ (Squire and Zola-Morgan, 1988). Memory systems which fell under this classification were defined as memories that could be expressed through performance rather than recollection. On the other hand, memories that appeared to be impaired in hippocampal amnesia were ‘declarative’ in nature (Cohen and Squire, 1980), that is, they involved the conscious recollection of facts and events. Moreover, and unlike nondeclarative memories, declarative memory was considered to enable facts and events to be represented in highly linked relational and flexible networks (Cohen, 1984), a flexibility that was purported to permit these memories to be consciously accessed and “declared” (Konkel and Cohen, 2009). Although this was not the only theoretical account proposed at the time, all extant views embodied a similar outlook, i.e. that hippocampal damage compromises some mnemonic processes while others are spared, and that the memory system that is impaired involves the representation of memories that are within conscious awareness (or explicit memory; Graf and Schacter, 1985). Such ideas went on to dominant memory research in the adult literature for many decades (they have been questioned in recent years – see Maguire and Mullally, 2013), largely because it provided a useful biological framework by which memory performance, across an array of different tasks, populations and species could be classified using a similar set of concrete criteria (Squire, 2004).

### 2.1 Multiple memory systems in infants

It is not surprising then that at this time in the 1980s memory researchers began to ask whether similar dissociations could be observed in infant memory (Nadel and Zola-Morgan, 1984, Schacter and Moscovitch, 1984). Such questions came on the back of numerous studies conducted in the 1970s and early 1980s which started to challenge the then prevailing view of infant memory, namely that infants lacked the capacity for long-term memory. These studies presented clear evidence that even in the initial hours and days after birth, infants were capable of learning and expressing knowledge across long periods of time (Rovee-Collier and Fagen, 1981). However, these studies often lacked theoretical focus (Fagan, 1984; for exceptions see Cohen and Gelber, 1975, Olsen, 1976) and their findings were typically “accounted for by invoking a unitary concept of memory” (Schacter and Moscovitch, 1984, p. 174). Guided by the current thinking, memory researchers examined the infant literature to see if the apparent absence of certain forms of memory in infants, coupled with demonstrations of quite strikingly intact memory, could be explained within the multiple memory system biological framework being advocated at the time.

For example, Schacter and Moscovitch (1984) proposed that infants have two memory systems; an early system that corresponds to nondeclarative memory (spared following hippocampal damage) that is available to infants from birth, and a later system that corresponds to declarative memory (impaired following hippocampal damage) that is available in the latter part of an infant's first year (at around 8–9 months of age; Kagan and Hamburg, 1981). The authors argued that the visual paired comparison task (VPC; also referred to as habituation and novelty-preference paradigm) typically used to assess infant memory (where infants spend less time looking at a familiar relative to a novel stimulus) actually tapped into unconscious memory, and therefore did not provide evidence of early hippocampal-dependent memory. Schacter and Moscovitch (1984) suggested that this was because successful performance on such paradigms occurs as a result of modifications of perceptual-cognitive processes (i.e. priming processes) without any explicit knowledge of, or any explicit reference to, the study context.

While this proposal made theoretical sense, it is very difficult, if not impossible, to ascertain whether pre-linguistic infants have conscious awareness of the study context or not (Nelson, 1997, Rovee-Collier, 1997, Rovee-Collier and Cuevas, 2009). Given that this is the critical dimension upon which the multiple memory systems account resides, it is necessary to look for other ways in which infant tasks can be accurately classified. In order to achieve this, Schacter and Moscovitch (1984) proposed what later became known as the ‘parameter’ filter. The logic here is that as performance on tasks of declarative memory is typically influenced by variables such as retention interval, study duration and context changes, whereas performance on nondeclarative tasks is not, then identifying whether a task is impacted by the manipulation of these variables can assist the classification of the task as either declarative or nondeclarative (Hayne, 2007b). Schacter and Moscovitch (1984) reviewed data from numerous VPC studies available at the time and concluded that variables such as retention interval did not appear to alter infants’ performance (e.g. Fagan, 1971, Fagan, 1973). This resistance to the impact of increasing the retention interval was interpreted as evidence of the nondeclarative nature of the tasks. This, therefore, supported the proposal that the early mnemonic achievements of young infants are attributable to an early, non-hippocampal-dependent, memory system.

What of the suggested late memory system? Adopting the parameter filter again, Schacter and Moscovitch (1984) argued that one could examine the effect of modality shifts between study and test, a manipulation that is often detrimental to performance on nondeclarative priming tasks, but which does not typically impact performance on declarative memory tasks, to garner such evidence. Interestingly, novelty preference had been shown to be eliminated in young infants (6- to 9-months) via a cross modal switch between study and test, an impairment which is not evident in older infants (12-months; Gottfried et al., 1977), when the late (declarative) memory system has matured sufficiently to support this switch. Thus, tentative evidence appeared to exist in favour of this early/late memory systems account of infant memory.

**Fig. 3:**
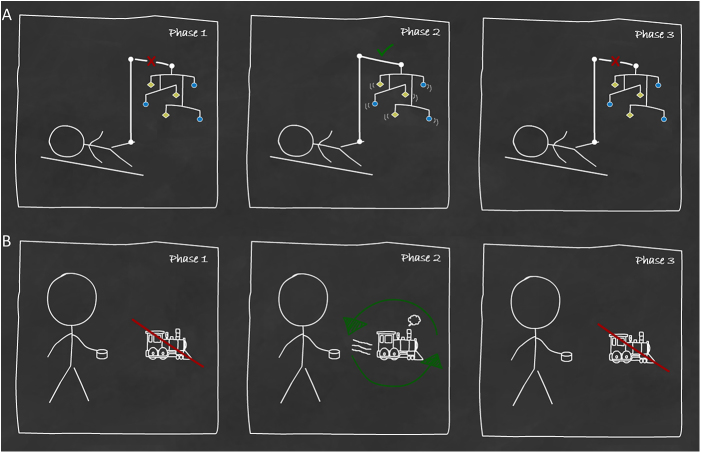
The operant conditioning paradigms. (A) The mobile conjugate reinforcement paradigm (Rovee-Collier et al., 1980; suitable for use in 2–7 month old infants). The left panel illustrates phase 1: the baseline condition. Here the ankle ribbon is not connected to the mobile so that when the infant kicks they do not move the mobile. The middle panel illustrates phase 2, the acquisition phase, where the ankle ribbon and the mobile are connected so that when the infant kicks, the mobile conjugately moves. The right panel illustrates phase 3, the retention phase. Here, as in phase 1, the ankle ribbon and the mobile are not connected. However, if the infant recognised the mobile, they should kick to move the mobile. Memory of the mobile is therefore indexed by an increased rate of kicking in phase 3 relative to phase 1. (B) The operant train task (Hartshorn and Rovee-Collier, 1997; suitable for use in 6–24 month old infants). As with the operant mobile task, phase 1 (left panel) provides a baseline measure. Here the lever is deactivated and therefore when the infant presses the lever the train does not move. In phase 2 (middle panel), each lever press made by the infant moves the toy train for 1 or 2 s (depending on the infant's age). In phase 3 (right panel–the retention phase) the lever is again deactivated and memory for the train is indexed by an increased rate of lever pressing relative to the baseline pressing rate in phase 1.

Since its proposal almost 30 years ago, this account, which is often referred to as the neuromaturational account (Rovee-Collier and Giles, 2010), has received much support and is still a dominant view in the field of infant memory (Bauer, 2006, Bauer, 2008). However, there is controversy surrounding the question of how tasks suitable for use in infants and young children are classified. Another benchmark of declarative memory that is commonly used is the ‘amnesia’ filter (Squire and Schacter, 2002), whereby a task is considered to be declarative (and hence hippocampal-dependent) if performance on the same task is impaired in adult patients with hippocampal amnesia. However, even with both the ‘parameter’ and the ‘amnesia’ filters in place, there is still debate about whether some of the key tasks used in the infant literature should be classified as declarative or nondeclarative. These key tasks include the VPC paradigms previously discussed, as well as operant conditioning (e.g. the mobile conjugate reinforcement paradigm – Fig. 3A, and the train paradigm – Fig. 3B; Rovee-Collier and Hayne, 2000) and imitation protocols. In the latter tasks, a demonstration of an action or sequence of actions is given and infants imitate this action either immediately (elicited imitation), or after a delay period (deferred imitation).

As with the VPC, Schacter and Moscovitch (1984) argued that the operant conditioning paradigm devised by Rovee-Collier and Fagen (1981; Fig. 3) is nondeclarative in nature and thus dismissed the early findings of advanced mnemonic function in 3- to 5-month-old infants (Rovee-Collier et al., 1980, Rovee-Collier and Fagen, 1981). Similar concerns regarding the operant conditioning paradigm (and in particular the mobile conjugate reinforcement, Fig. 3A) have been expressed by others (Bauer, 2008) who argue that such tasks most likely depend upon the cerebellum and deep nuclei of the brain stem, which mature earlier than the hippocampus, and which most likely support a primitive, nondeclarative memory system. However, using the parameter filter, variables such as age, retention interval, study time and context changes have all been found to influence performance on the VPC, tasks of operant conditioning and imitation procedures (Hayne, 2004, Rovee-Collier, 1997), suggesting these tasks should be considered declarative in nature. Similarly, variables that influence adults’ performance on declarative memory tasks, such as interference, levels of processing and serial position, also impact infants’ performance on the deferred imitation task (Hayne, 2007b), leading to the same conclusion, that all three of these paradigms appear to tap into declarative, as opposed to nondeclarative, processes (Hayne, 2004).

The use of the amnesia filter as a tool for classifying these tasks also indicates that the VPC (McKee and Squire, 1993, Pascalis et al., 2004) and deferred imitation (McDonough et al., 1995, Adlam et al., 2005) paradigms are hippocampal-dependent and should be classified as declarative (Nelson, 1995). Of note, the amnesia filter is agnostic with respect to the classification of the operant conditioning task because the mobile task is unsuitable for use in adult populations (or children over 6 months of age – see Fig. 3), and after the age of 2 years, participants simply cease performing the train task, declaring to the experimenter in phase 3 that the train is broken, or that the batteries need to be replaced (Hildreth and Hill, 2003, Hsu and Rovee-Collier, 2006). Interestingly, however, Gross et al. (2002) reported identical results when 6-month-old infants were tested on both operant and imitation tasks, suggesting that these two measures may tap into the same underlying function.

### 2.2 The emergence of declarative memory

**Fig. 4:**
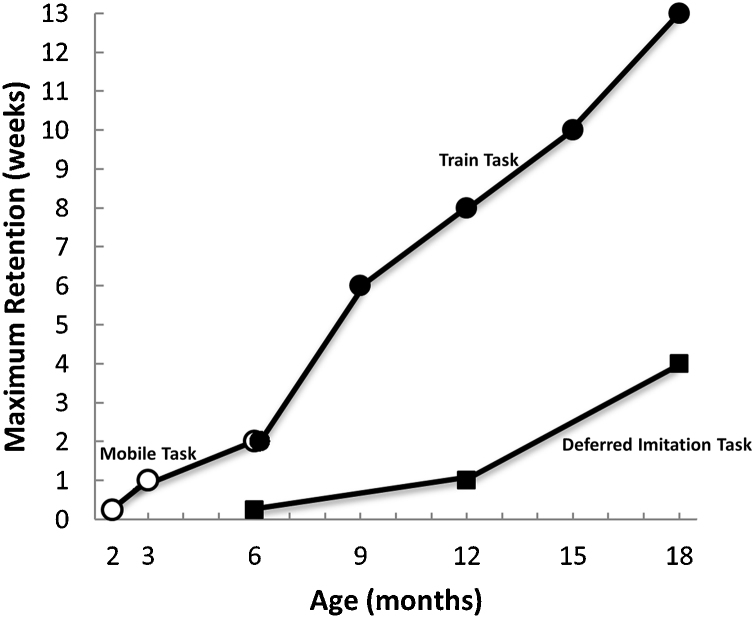
Standardised reference functions for the maximum duration retention of infants on the operant mobile, operant train and deferred imitation puppet tasks. Maximum retention duration (*x*-axis) appears to increase linearly as a function of increasing age (*y*-axis). Note that the difference in the slope of the two functions is attributed to the different training parameters used in these paradigms. This graph has been redrawn exactly from Rovee-Collier and Cuevas (2009), and is reprinted with permission from the American Psychological Association.

If one accepts that these tasks tap into one underlying memory system, then the key question is when does the memory system that supports these tasks become functional? Hayne (2007b) has argued that across multiple laboratories and studies using the VPC, operant conditioning or imitation paradigms, consistent patterns have emerged which can be summarised in terms of three general principles (Hayne, 2004). First, *older infants encode information faster than younger infants*. For example, using a VPC task, Fantz (1964) found that 3- to 4-month-old infants needed more exposure to the familiar stimulus in order to demonstrate a novelty preference than 4- to 6-month-old infants, and Rose (1983) reported that 6-month-old infants required a 15 s exposure to the familiarisation stimulus, but that by 12 months this time had decreased to 10 s. Similarly, on the mobile conjugate reinforcement task, 2-month-old infants typically learned the task within 3–6 min (Davis and Rovee-Collier, 1983), 3-month-old infants learned within 2–3 min (Greco et al., 1986), and 6-month-old infants within 1 min (Hill et al., 1988); and on a task of deferred imitation, 6-month-old infants required twice as much exposure to the target actions than older infants (12-, 18-, and 24-month-olds; Barr et al., 1996). Thus, increasing age appears to correspond to a shortening of encoding times across a range of mnemonic paradigms. Second, *younger infants appear to remember for shorter periods of time*. Six-month-old infants imitated actions for only 24 h (Meltzoff, 1988), 9-month-olds recollected actions for up to 5 weeks (but not 3 months), while just 1 month later, 10-month-olds could reproduce the same actions for up to 3 months (Carver and Bauer, 2001; see also Fig. 4). Finally, *memory in younger infants is considered to be highly specific, with older children utilising a wider range of retrieval cues than their younger counterparts.* For example, in an imitation task, retrieval is easily disrupted by a change in the cues between encoding and test (Hayne, 2004, Rovee-Collier, 1997). In addition, Hayne et al. (2000) demonstrated that when either the form or the colour of a puppet was changed at test (relative to the original demonstration), 6- and 12- (but not 18-) month old infants’ performance at retrieval was disrupted, while a major change in context between the study and the test phases left 6- (but not 12- or 18-) month old infants impaired.

Evidently, and despite age-related changes across these three dimensions, declarative (or hippocampal-dependent) memory appears to be evident by the middle of the first year and perhaps even earlier (Hayne, 2007b), which is long before the age originally suggested by Schacter and Moscovitch (1984) and advocated more recently by others (Bauer, 2006, Bauer, 2008). Moreover, principles 1 and 2 do not necessarily appear to provide evidence of an early (or primitive) memory system which gives way to a late, more sophisticated, declarative memory system. Rather these findings could simply be indicative of a memory system (which we will, for now, continue to refer to as a declarative memory system) that is rapidly developing and increases monotonically throughout infancy but which, during this development, is fragile and less efficient. Fig. 4 illustrates the linearity between observed retention duration (on the operant mobile and train tasks and the deferred imitation task) and age. No sudden shifts in retention duration are evident at any age point, which would presumably be anticipated if a sudden shift between memory systems was occurring. This unitary interpretation is supported by the work of Rovee-Collier and colleagues who argue that, given sufficient support, the enduring nature of infant memory can be enhanced. For instance, 2-month-olds typically forget the mobile conjugate reinforcement task (Fig. 3A) within 1–2 days. However, retention can be extended if the infant is exposed to regular reminders every 3 weeks (Rovee-Collier et al., 1999). In fact, in this study, retention was still evident at 7.25 months, a full 4.5 months after initial acquisition, at which point it could be assessed no further due to the inappropriate nature of the task for older children. Critically, a control group who were exposed to the same reminders but not the initial acquisition showed no retention, ensuring that it was the task, and not the reminders of the task, that was being recalled at test. Furthermore, in a follow-up study in older children (which therefore used the operant conditioning train paradigm), acquisition of the task occurred when infants were 6-months-old, followed by five spaced reminders in the intervening period (with the final reminder occurring at 18-months of age). This resulted in the infants (now 2 years of age) exhibiting significant retention of the task 1.5 years after acquisition (Hartshorn, 2003). Therefore, very young infants (i.e. 2- or 6-month-olds) appear capable of forming enduring memories provided the retention of those memories is given sufficient support. Why these memories require such support remains an open question.

**Fig. 5:**
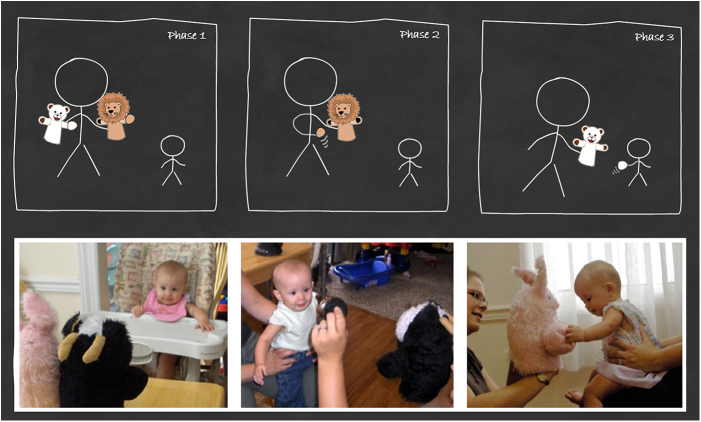
Sensory preconditioning and the deferred imitation puppet task (Barr et al., 2003). The left panel illustrates the sensory preconditioning whereby an infant receives paired pre-exposure to puppet A and puppet B. In phase 2 (middle panel) the target actions (remove the mitten from the puppet's hand, shake the mitten, replace the mitten) were demonstrated for the infant on puppet A. The deferred imitation test then occurs in phase 3 (right panel). Here the memory of the pairing between puppet A and puppet B is demonstrated if the infant models the target actions on puppet B. This phase can only be performed by infants aged 6 months and above, as younger infants are motorically incapable of performing the target actions themselves. Lower panels reproduced from Rovee-Collier and Giles (2010) with the permission of Elsevier.

Notably, similar findings have been observed using tasks of deferred imitation, which are accepted by many to tap into the declarative memory system (but see Newcombe et al., 2012). For instance, the retention duration of a multi-step sequence (remove the mitten from the puppet's hand, shake the mitten, replace the mitten; see Fig. 5) can be increased by manipulating a number of factors. Barr et al. (2005) observed deferred imitation for at least 10 weeks after the initial exposure in 6-month-old infants, provided the infants retrieved the memory of the sequence of actions on multiple occasions within this 10-week period and that these retrievals were widely spaced out in time. Similarly, Campanella and Rovee-Collier (2005) found that 3-month-old infants retained and imitated modelled actions when retention was tested 3 months after acquisition, when the infants were now 6-months-old, provided the memory of the actions had been periodically reactivated in the intervening time period. Thus, enduring ordered recall is evident in very young infants and these data (plus the results of the operant conditioning tasks discussed above) appear to demand a radical shift in how we conceptualise the mnemonic capabilities of very young children. Moreover, they beg questions of the neuroscientific community to provide explanations of how these memory traces are being acquired and consolidated in such immature brains.

### 2.3 The emergence of episodic memory

Until now we have used the term declarative memory to refer to memories that depend upon the hippocampus. This is largely because ‘declarative’ memory and ‘hippocampal-dependent’ memory were once viewed as synonymous and, although controversial, declarative memory (via the use of the parameter and amnesia filters) was relatively easy to assess in infants, as described earlier. However, it is highly likely that there is more to hippocampal-dependent memory than declarative processes, and in recent years the type of memory that has been most associated with the hippocampus is episodic memory – the memory for our personal past experiences. Hence, in order to be truly convinced that young infants are utilising a hippocampal-dependent memory system then we need to also look for evidence of episodic memory in this population.

Some have argued that children under the age of 4- or 5-years of age are unable to form episodic memories (e.g. Tulving, 2005). Because episodic memory is difficult (if not impossible) to assess in the absence of language (Clayton et al., 2003), such studies often utilise ‘episodic-like’ memory paradigms (for a review see Salwiczek et al., 2010) which attempt to measure the recollection of “happenings in particular places at particular times” (Tulving, 2002, p. 3), or the ‘what-where-when’ (www) of episodic memory (Tulving, 1972). While episodic-like memory (or ‘www-memory’) paradigms have been successfully utilised across a range of nonhuman species (Clayton and Dickinson, 1998, Ergorul and Eichenbaum, 2004, Martin-Ordas et al., 2010), more recent studies have employed this approach to track the emergence of episodic memory across childhood. For instance, Hayne and Imuta (2011) developed a hide-and-seek paradigm to assess young children's (3- and 4-year-olds) ability to recollect the ‘www’ of a hiding event, while Bauer et al. (2012) focused their attention on identifying the point at which the ‘where’ of personally experienced events is successfully bound with the event details themselves. In keeping with previous hypotheses (Tulving, 2002, Tulving, 2005), this form of memory appears to show a protracted development throughout early and middle childhood, although rudimentary episodic memory skills do appear to be in place by the age of 3 (Hayne and Imuta, 2011). Interestingly, more recent findings have suggested that it is the ability to retain, as opposed to form, episodic memories that may be the source of the advantage inferred through age in older children, with 3-year-old children demonstrating good retention of episodic recollection across short but not long delays (Scarf et al., 2013).

Interestingly, the ‘www-memory’ approach has firmly aligned itself with the associative/relational account of episodic memory (for review see Eichenbaum, 2004). Under this model, episodic memory processes are considered to involve the binding together of disparate pieces of information (the what, where and when) to form a sequentially organised, novel, associative representation of the event in question. This associative representation does not, however, occur in isolation; instead it overlaps with other associative representations that share a common element (e.g. with episodic memories that have occurred in the same location). In this way, common elements serve to organise episodic memories into relational networks, linking new and existing memories to one another. Such networks enable the individual to compare and contrast memories, to make inferences among indirectly related episodic events, and even to form expectations about future experiences. It is this flexibility, ultimately afforded by the associative nature of episodic memory, that is considered by many to be a fundamental feature of hippocampal-dependent memory (Eichenbaum, 2004). Hence, it can be appreciated why many developmental scientists consider that at its heart, the development of episodic memory “is the development of the ability to bind together information co-occurring at a particular time in a particular spatial context” (Newcombe et al., 2012, p. 75) and why relational memory is considered to be a canonical form of episodic memory (Koski et al., 2013). The question is, therefore, at what age do basic associative/relational processes become apparent, and can we use this as evidence of episodic-like memory abilities in infancy?

**Fig. 6:**
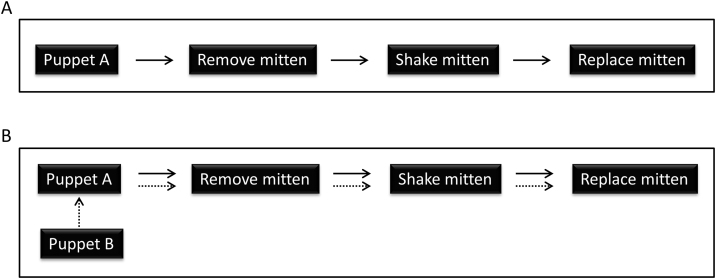
(A) The hypothesised associative representation containing each element of the event sequence depicted in Fig. 5 – the deferred imitation puppet task. (B) Here, this associative representation has been subsumed into a larger relational network which also contains the association between puppet A and puppet B (encoded by the infants during the sensory pre-conditioning phase), which subsequently enables the infant to transfer context-specific associations (associations bound to puppet A) across contexts (to puppet B).

As previously discussed, one characteristic of infants’ memory performance on the tasks described earlier is that it appears to be highly specific and inflexible (Hayne, 2004, principle 3 above), and hence incompatible with the above characterisation of episodic memory. However, recent demonstrations suggest that under certain conditions, and contrary to these earlier findings, very young infants can (1) form spontaneous and enduring associations, and (2) are capable of using these associative representations in a flexible manner. For instance, using the mobile conjugate reinforcement paradigm in combination with a sensory pre-conditioning phase (where the infants were pre-exposed to the study and test contexts, which in this case were two distinctive cloth panels), Boller (1997) found that 6-month-old infants continued to show significant retention of the mobile task (Fig. 3) despite a change in context between study and test. Critically, this was only observed for the infants who were pre-exposed to the cloth panels simultaneously; with the typical pattern of impairment evident in the control infants who had been pre-exposed to the two contexts individually but not simultaneously. Thus, the pre-exposed infants seemed to spontaneously form a lasting association between the contexts in the sensory pre-conditioning phase, and this associative representation appeared sufficient to enable them to demonstrate a flexibility often assumed to be absent in young infants’ memory. Similarly, Barr et al. (2003) pre-conditioned 6-month-old infants to two hand puppets (A and B; see Fig. 5A, left panel) using a similar simultaneous presentation technique. The infants then observed a sequence of three target actions (i.e. remove the mitten from the puppet's hand, shake the mitten, replace the mitten) demonstrated on puppet A (Fig. 5A, middle panel). Twenty-four hours later this group of infants spontaneously imitated these actions on puppet B (Fig. 5A, right panel) thus demonstrating a transfer of knowledge from puppet A to puppet B. Critically however, infants who had the same amount of pre-exposure to puppets A and B, but not simultaneous pre-exposure, did not model the actions on puppet B, suggesting these control infants had not formed an association between puppets A and B, and that the absence of this association rendered the memory isolated and non-transferable. Importantly, the specificity demonstrated by the control infants (and those in previous studies, e.g. Hayne et al., 2000), in tandem with the flexibility demonstrated by the experimental infants, argues against the suggestion that infants under the age of 2 years form only generalised or semantic representations of event sequences (Newcombe et al., 2012). This is because the above pattern of results demands that both groups’ recollection of the original event sequence must necessarily have contained specific item details, i.e. the identity of puppet A (Fig. 6). Hence, it is plausible that these infants formed an associative representation of the event sequences, which in the case of the experimental group, was subsumed into a larger relational network that also included the association between puppet A and puppet B (Fig. 6B). Thus, these fundamental associative elements of episodic memory may in fact be present in 6-month-old infants.

Interestingly, spontaneous associative learning is also evident in even younger infants. For instance, Campanella and Rovee-Collier (2005) found that 6-month-old infants spontaneously imitated target actions on puppet B, even though the simultaneous pre-exposure to the puppet pair (i.e. to puppets A and B), and the modelling of the target actions on puppet A, had occurred 3 months earlier, when the infants were just 3-months-old. The transfer of learning from puppet A to puppet B observed here occurred in spite of a 3-month delay between the sensory pre-conditioning phase, where the association between the puppet A and puppet B was learned, and the test phase (note, memory of the target actions was periodically reactivated with puppet A during this time). As before, the infants who had sequential but not simultaneous pre-exposure to puppets A and B did not model the actions on puppet B in phase 3 despite the fact that they (like the simultaneously pre-exposed group) had observed the target actions performed on puppet A on multiple occasions.

These results demonstrate that even 3-month-old infants seem capable of forming spontaneous associations between simultaneously occurring events and appear to use this associative knowledge flexibly in a novel context. But do these infants also form associations between items that have never been previously encountered together which, as discussed above, is often considered a key feature of a flexible memory system (Eichenbaum, 1997, Squire and Kandel, 1999)? Tasks where associations between indirectly related stimuli must be inferred are known as transitive inference tasks and the acquisition of transitive inferences was once considered to emerge around 7 years of age (Piaget, 1928, Townsend et al., 2010). Cuevas et al. (2006), however, tested whether such flexibility could be demonstrated in 6-month-old infants.

Here, the infants were simultaneously exposed to puppets A and B (phase 1: association between puppet A and B presumed to be formed) and then trained to kick a mobile in a distinctive context 24 h later (phase 2: association between mobile and context presumed to be formed). A third phase then ensued where the infants were exposed to puppet A (without the presence of puppet B) in the distinctive context (without the presence of the mobile). Thus in phase 3, the presence of puppet A was presumed to activate the associated memory of puppet B, while the distinctive context was presumed to activate the associated memory of the mobile. The question was whether this would lead to the formation of a new association between the co-activated memory representations (puppet B and the mobile) despite the fact that neither puppet B nor the mobile were ever physically presented together. Interestingly, this was what Cuevas et al. (2006) found. Moreover, this association appeared to persist for up to 2 weeks. Therefore, under certain conditions, very young infants’ memory appears to be far from rigid and specific.

However, what about the previously-described findings that failed to find evidence of flexibility in infant memory (e.g. Hayne et al., 2000)? One explanation proposes that it is infants’ lack of world knowledge and experience (Rovee-Collier and Cuevas, 2009) that is the source of this apparent inflexibility. For example, Richmond and Nelson (2007) proposed that the inclusion of a sensory pre-conditioning phase where the infants learned about the relationship between two items (e.g. between puppet A and puppet B), provides the prior knowledge, or relational network, into which the novel event (or associative representation) can be embedded. This then enables the infant to demonstrate these surprisingly advanced forms of relational memory. Hence, in experimental contexts where this support is absent, infants may lack a sufficiently rich network of knowledge within which to integrate the event, and the absence of this knowledge-based network renders the memory isolated and inflexible (Barr et al., 2011). In support of this idea, Richmond and Nelson (2007) cited the observation that 9-month-old infants who are able to crawl and likely acquire a richer representation of their environment (or the ‘where’ of their episodic memories) than their non-crawling counterparts appear to be more capable of using their memory in a flexible manner than infants who are not yet crawling (Herbert et al., 2007). In a similar vein, Jones et al. (2011) sought to determine whether the limitations in representational flexibility (i.e. the failure to recognise previously viewed stimuli when presented on a new background) observed in infants younger than 18-months by Robinson and Pascalis (2004) could be overcome by the provision of variability training during encoding (i.e. the presentation of the studied item on multiple backgrounds). Significantly, they demonstrated that this training enabled infants as young as 6-months-old to recognise the studied item when it was subsequently presented on a novel background. Such findings are consistent with the proposal that an apparent inflexibility in infants’ memory may be driven by their lack of world knowledge and experience, and not necessarily by an absence of a flexible associative/relational memory ability.

### 2.4 A transitional age in the ontogeny of episodic memory?

While there is evidence that very young infants may be capable of impressive mnemonic feats (including flexibility), it is perhaps noteworthy that infants begin to crawl and hence gain the associated cognitive benefits of independent locomotion at ∼9 months. This is interesting as 9-months has been consistently described by memory theorists as a critical transitional age whereby infant memory appears to undergo a radical development. As previously described, many have argued that at this age one memory system (an early developing nondeclarative memory system) is supplanted by another (a late developing declarative memory system; Schacter and Moscovitch, 1984, Bauer, 2006, Bauer, 2008), or a pre-explicit memory system is supplanted by an explicit one (Richmond and Nelson, 2007), while others have described this as the age at which the period of exuberant associative learning observed in early infanthood appears to come to an end (Rovee-Collier and Giles, 2010). Additional evidence that this represents an important developmental age in the ontogeny of hippocampal-dependent memory comes from the work of Bauer and colleagues (Carver et al., 2000). They found that 9-month-old infants who successfully imitated previously-learned event sequences also showed brain activity patterns consistent with recollection (i.e. a late positive slow wave component of the ERP response; Paller and Kutas, 1992), which was lacking in the 9-month-old infants who did not imitate (Carver et al., 2000) and in amnesic patients (Düzel et al., 2001, Addante et al., 2012). Richmond and Nelson (2009) recently utilised an eye-tracking technique (previously employed to investigate relational memory in adult amnesia; Hannula et al., 2007), to demonstrate that 9-month-old infants can encode the relations among items (i.e. between novel faces superimposed on unique scenic backgrounds; but see also Koski et al., 2013). However, it is unclear from these data sets whether infants below the age of 9-months would also demonstrate a late positive slow wave (ERP) or show evidence of successful relational encoding (eye tracking).

Nevertheless, the fact that Herbert et al.’s (2007) findings tentatively linked the emergence of flexible memory to the onset of independent locomotion resonates with a hypothesis proposed by Nadel and Zola-Morgan (1984). They argued that true episodic memory (that encapsulates the ‘when’ and ‘where’ of the recollected episode) will only be possible once an organism is capable of hippocampal-dependent place learning (i.e. allocentric spatial learning). In support of this they cited findings from the animal literature demonstrating that place learning appears quite abruptly on postnatal day 19 in the life of a rat (e.g. Sutherland, 1982, personal communication; cited in Nadel and Zola-Morgan, 1984), and linked this form of learning with the emergence of the hippocampal memory system. This coincides with more recent observations that adult-like grid cells first emerge on postnatal day 19 (Wills et al., 2012; see also Wills et al., 2010, Langston et al., 2010) with all the basic components of a rat's hippocampal navigation system in place by 3 weeks of age, which is also the age at which weanlings first start to leave their nest (Gerrish and Alberts, 1996) to begin independent spatial exploration. (Note: this is also the age at which the effects of rat infantile amnesia begin to dissipate, with infantile amnesia appearing strongest in rat pups <21-days-old; for review see Callaghan et al., 2014). Prior to the emergence of this place learning, Nadel and Zola-Morgan (1984) argued that although the experiencing of events will influence an organism (and ‘leave behind some residue’), true “in place” memory will not be evident, which resonates with the hypothesis that prior to the advent of crawling in a human infant's life, memory appears to be inflexible and somewhat improvised. In this way, independent locomotion may herald the development of more sophisticated spatial knowledge, which in turn may provide the scaffolding (the “in place” or the “where” component of episodic memory) necessary to support flexible and complex recollections of past experiences.

Returning to the idea that 9-months of age represents the end of an period of exuberant associative learning, Reynolds and Rovee-Collier (2005, cited in Rovee-Collier and Giles, 2010) found that both 6- and 9-month-old infants who were simultaneously pre-exposed to puppet A and puppet B (Fig. 5), and who observed target actions performed on puppet A, recollected and performed the modelled actions on puppet B after a 2 (but not a three) week delay. Twelve-month-old infants however failed to demonstrate the actions after any delay. This raises the interesting possibility that very young infants may actually form more spontaneous associations and retain these associations for longer time periods than older infants. Similarly, Cuevas et al. (2009; cited in Rovee-Collier and Giles, 2010) found that while 6-month-old infants associated the two puppets following simultaneous but not sequential pre-exposure, 9-month-old infants associated the puppets after either simultaneous or sequential pre-exposure, but 12-month-old infants only associated puppets that had been sequentially, but not simultaneously, presented. This suggests that there is a change in what infants spontaneously associate before and after this critical age period. This has led some to propose that the exuberant learning which appears to occur in very early infancy spontaneously ends around the transitional age of 9 months (Rovee-Collier and Giles, 2010). Understanding the neural events that occur prior to, during, and after this transitional period is over, could shed important light on the neural substrates underpinning both of these phases and help to address the question of whether this represents a fundamental shift between memory systems or an incremental change within the infants’ fledgling episodic memory system. Regardless, the above findings suggest that very young infants are potentially utilising a more sophisticated form of memory than many theories of early memory development would suggest.

How close do these data move us towards understanding whether very young infants have a functional episodic memory system? In other words, is evidence that very young infants are capable of forming and recollecting complex relational memories akin to providing evidence that these infants are forming and subsequently recollecting complex episodic memories? Many would argue that this is not sufficient. For instance, the question of whether successful performance on a ‘www-memory’ paradigm is analogous to true episodic memory has been fiercely debated in the literature (for review see Salwiczek et al., 2010). Moreover, Tulving's original ‘www-definition’ of episodic memory (Tulving, 1972) has been updated a number of times so that it now includes a conscious awareness that an event is ‘remembered’ as opposed to being simply familiar or ‘known’ (autonoetic consciousness; Tulving, 1985), and an ability to use episodic memory to project oneself into both the past and future (chronesthesia; Tulving, 2002). Strict adherence to such a complex and linguistically-dependent definition of episodic memory makes establishing whether episodic memory defined along these dimensions is present in very young infants almost impossible (although a number of researchers have attempted to explore it in children aged 3-years and older; e.g. Scarf et al., 2013, Russell et al., 2010, Busby and Suddendorf, 2005). Can any evidence of a functioning episodic memory system be gathered by studying children's earliest memories? And if such evidence is present, how can the above reports of flexible associative memory in early infancy be reconciled with the phenomenon of infantile amnesia?

## 3 Children's earliest memories

The offset of infantile amnesia occurs between 3 and 5 years of age, and this offset is often considered to herald the onset of a memory system capable of supporting enduring episodic memories. Support for this comes from a century of empirical evidence. In one such study, college students were asked to recollect childhood memories of significant early life events (such as the birth of a sibling, the death of a close relative, a period of hospitalisation; Usher and Neisser, 1993). Despite the dramatic nature of these events, participants rarely reported any memories prior to the age of two, and only incomplete memories until the age of four, and thus conformed to most adults’ subjective experience of infantile amnesia. However, there is an important caveat in the infantile amnesia literature in that most studies have been conducted in adult populations. Such studies cannot therefore address the question of whether children themselves experience the same degree of infantile amnesia. In respect of this, Sheingold and Teeney (1982) found that young children recalled little of a sibling's birth if this birth occurred prior to their 4th birthday, and this did not appear to be related to the participant's age at the time of testing (which ranged from 4- to 12-years of age). This appeared to confirm the findings of studies conducted in adults.

However, a different picture begins to emerge when children's earliest episodic memories are specifically targeted. Peterson et al. (2005) found evidence to suggest that the age of a child's earliest episodic memory is influenced by that child's current age, with younger children appearing capable of recollecting memories from significantly younger ages than older children. Similarly, in a recent and comprehensive investigation of the offset of childhood amnesia in developmental populations, Tustin and Hayne (2010) found that the earliest memories accurately reported by children and adolescents occurred at significantly younger ages than the traditional 3.5-year-old boundary of infantile amnesia observed in adults. In fact, both groups of children, who were either 5-years-old or 8- to 9-years-old at the time of testing, had an average age for their earliest memory of less than 2 years, and in 12- to 13-year-olds this rose slightly to 2.5-years of age. Interestingly, in the same study, Tustin and Hayne (2010) also elicited early memories of episodic events that had occurred prior to their participants’ 3rd birthdays. When these data were combined, the earliest memory of some children, who were under the age of 10 at the time of testing, was for events that had occurred when they were less than 1-year-old. In fact, memories of episodic events occurring between birth and 1-year of age accounted for over 20% of all the early memories given by these children. When inspected further, all but one of these very early memories occurred before the age of 6-months (see Table 1; Tustin and Hayne, personal communication). More striking still was the observation that 50% of these very early memories were of events that had occurred when the children in question were less than 1-month-old. These memories were accurate (verified by a parent) and episodic in nature. These findings, albeit from one study, therefore suggest that extremely young infants are potentially capable of encoding a proportion of their personal experiences from birth, and that at least some of these memories can be later recollected in an episodic manner up to 8- or 9-years later, potentially challenging the previous theoretical accounts of infant memory (see also Jack et al., 2012).

**Table 1:** Distribution of early memories from the 0 to 12 months of age time bracket.

	Participant age				
Age of memory	5-years	8–9 years	12–13 years	18–20 years	Total
0–1 months		4			4
1–2 months					
2–3 months	1	1			2
3–4 months					
4–5 months					
5–6 months	1				1
6–7 months					
7–8 months					
8–9 months					
9–10 months					
10–11 months					
11–12 months	1				1
Total	3	5	0	0	8

Tustin and Hayne, personal communication; taken from Tustin and Hayne (2010).

Of the 8 early memories that were recollected by participants, all but one occurred prior to the age of 6-months of age. Significantly, these very early memories were only provided by the two youngest age groups tested (the 5 year olds and the 8–9 year olds). The earliest memories provided by participants in the two older groups (the 12–13 year olds and the 18–20 year olds) occurred between their 1st and 2nd years of life and hence they reported no memories within the above time frame.

Why these memories are sparse and why after the age of 10 these memories then become inaccessible or forgotten are intriguing questions. One possibility is that very early episodic memories are not encoded as successfully or as completely as later memories (Tustin and Hayne, 2010). While this fits with a model of episodic memory that does not become fully functional until late into the first decade of life (Bauer et al., 2012), it does not accord with findings from rodent models of infantile amnesia, whereby rats demonstrate profoundly impaired long-term memory for events occurring in early life (<21 days) despite having previously demonstrated successful initial encoding (for a review see Callaghan et al., 2014). It is thus possible that human infants are successfully encoding the events of their early lives in an episodic manner, but that due to insufficient or disrupted consolidation processes, these memories are latter inaccessible. Why some of these memories remain accessible in some children for almost a decade before succumbing to what seems like an inevitable fate, represents an intriguing avenue of episodic memory research and is one which could potentially offer mechanistic insights into how episodic memories are successfully encoded, maintained and later recollected across a lifetime.

Interestingly, the neuroscientific community have very recently begun to recognise the potential of acquiring an in-depth understanding of the phenomenon of infantile amnesia. For instance, Josselyn and Frankland (2012) presented a novel proposal – the neurogenic hypothesis – which argues that despite normal (or near normal) initial memory formation in the infant brain, the integration of new neurons (neurogenesis) during development subsequently degrades the memory either by increasing the excitability of the hippocampal memory network or by replacing synaptic connections in pre-existing hippocampal circuits. As such, this theory essentially asserts that the high levels of hippocampal neurogenesis renders “all hippocampal memories destined to fade as they succumb to neurogenesis-induced decay” (Frankland et al., 2013, p. 5). This hypothesis can potentially account for why young children may retain the ability to recollect some very early memories while older children and adults cannot (see Callaghan et al., 2014, for a description of alternative molecular mechanisms underlying infantile amnesia). Considering the complex way in which human episodic memory is often defined, perhaps elucidation of its early ontogeny cannot be achieved through behavioural testing alone. Drawing inspiration from these neuroscientific explanations of infantile amnesia, we wonder if a full understanding of episodic memory throughout infancy and childhood will only be forthcoming when the development of the neural system supporting these functions is also taken into account.

## 4 Anatomical development

### 4.1 The structural maturation of the hippocampus

Theories advocating the delayed emergence of a hippocampal-dependent memory system were initially supported by anatomical findings in rats. Infant rats have very immature MTL structures and this was assumed to be also true of human infants (Bachevalier, 1992). But hippocampal development in human infants is actually more advanced (Seress, 2001), with only 15%, as opposed to 85%, of granule cells in the dentate gyrus being formed postnatally in the primate relative to the rodent hippocampus (Bayer, 1980, Rakic and Nowakowski, 1981). However, this protracted postnatal development of the cytoarchitecture of the dentate gyrus (the major route into the hippocampus) and a delayed maturation of hippocampal inhibitory interneurons (Seress et al., 1993), have led some to suggest that true hippocampal-dependent adult-like memory should not be expected earlier than 3–5 years of age (Seress and Abraham, 2008, Seress, 2001, Huttenlocher and Dabholkar, 1997, Lavenex and Banta Lavenex, 2013), an age that corresponds with that accepted for the offset of infantile amnesia.

Interestingly, in a systematic study of the structural and molecular changes that occur postnatally in the hippocampal formation of the rhesus macaque monkey, Lavenex and Banta Lavenex (2013) identified three distinct hippocampal circuits that appear to show a differential rate of postnatal maturation. First, and consistent with previous observations, Lavenex and Banta Lavenex observed a protracted development in the dentate gyrus (which they propose may persist for the first decade of human life), and an accompanied late development of specific layers located downstream of the dentate gyrus, particularly in the CA3 region. In contrast, they noted that distinct layers in several hippocampal regions that receive direct projections from the entorhinal cortex (such as CA1, CA2 and subiculum) appear to show early postnatal development. Moreover, the highly interconnected subcortical structures (subiculum, presubiculum, parasubiculum and CA2) seemed to develop even more rapidly, with tentative evidence of regressive events in the structural maturation of presubicular neurons. The culmination of these findings led the authors to conclude that “differential maturation of distinct hippocampal circuits might underlie the emergence and maturation of different ‘hippocampus-dependent’ memory processes, ultimately leading to the emergence of episodic memory concomitant with the maturation of all hippocampal circuits” (Lavenex and Banta Lavenex, 2013, p. 9).

### 4.2 The neural correlates of infant memory

It is therefore possible that young infants may acquire associative representations and flexible relational networks using the same ‘traditional’ associative learning mechanism used by adults, which are presumed to support episodic memory, and depend upon the hippocampus. If the human hippocampus matures in a similar way to the macaque, these memories’ vulnerability to long-term forgetting could be due to an incomplete functioning of the hippocampal circuitry. In other words, the continued benefit brought to memory with increasing age could be a consequence of the coming online of additional hippocampal circuits and the concomitant increasing cohesiveness of hippocampal function.

Alternatively, the absence of a fully integrated and functional hippocampus in early infancy could indicate that the complex associative processes that are taking place in early infancy are not in fact supported by the hippocampus. For example, this learning may potentially be supported by a “fast mapping” mechanism which permits the rapid acquisition of novel associations and which, unlike traditional associative learning mechanisms, does not appear to depend upon the hippocampus (Sharon et al., 2011). The term fast mapping was coined to describe infants’ rapid acquisition of words following brief exposure time (Carey and Bartlett, 1978), and since then it has been hypothesised to be the general learning mechanism (Markson and Bloom, 1997) via which very young infants can perform these mnemonic feats (Rovee-Collier and Giles, 2010). Another possibility is that the formation of these novel associations is supported by extra-hippocampal regions within the MTL, such as the perirhinal cortex, which have been shown to support the rapid encoding of novel item pairs into single compound (or unitised) units (Haskins et al., 2008). Importantly, this form of associative learning appears to support familiarity-based recognition, and not the rich contextually-bound recollection that underpins episodic memory (e.g. Quamme et al., 2007). Hence, determining the nature of the associations encoded by very young infants may be of critical importance in enhancing our understanding of the relationship between the ability to form complex associative memories in early childhood, and the very early ontogeny of episodic memory.

The issues alluded to above remain unresolved because we have never directly examined the functionality of the human infant hippocampus in vivo. Instead, the infant memory field has depended on drawing inferences about the functions of the developing hippocampus by using out-dated theoretical frameworks adapted from the adult literature, such as using the concept of consciousness, to determine whether a task taps into hippocampal-dependent memory or not. This strategy is problematic because there is every chance these frameworks are inadequate, and indeed there are numerous examples of hippocampal-dependent nondeclarative memory in the adult literature (e.g. Chun and Phelps, 1999, Ryan et al., 2000, Ryan and Cohen, 2004). This approach is less problematic in the adult literature because the field is actively working with these concepts while simultaneously pursuing biological evidence for or against these ideas. However, in the infant memory literature, where direct neuropsychological or neuroimaging evidence is rarely acquired, these concepts are essentially presented as the ground truth. One then takes a task, classifies it using tools like the ‘parameter’ and the ‘amnesia’ filters, and then uses its classification to inform about the underlying infant brain development. While inferring hippocampal functionality in the infant through cognitive testing and inference alone may have been appropriate three decades ago (when Schacter and Moscovitch, 1984, proposed their model), surely in this era of advanced, non-invasive neuroimaging techniques we should be attempting to ask more sophisticated and potentially more useful questions of this developing system. In this way, the infant memory literature could be unshackled from terms like declarative, explicit and conscious memory, terms which have caused theoretical divides within the literature. Moreover, when we attempt to address the question of the ontogeny of episodic memory, we run into similar problems, caused primarily by the demand placed on researchers to find evidence of complex cognitive processes considered to tap into the defining qualities of episodic memory, such as autonoetic consciousness (Tulving, 2005). Could many of these problems be mitigated by using neuroimaging techniques to directly study the emergence of episodic memory?

### 4.3 Neuroimaging the ontogeny of episodic memory

Of course, imaging infants and very young children using techniques such as MRI is not without its challenges (Raschle et al., 2012), especially if we wish to explore cognitive processing in the non-sedated infant. However, these challenges are not insurmountable. Functional MRI (fMRI) data have been successfully acquired in both non-sedated spontaneously sleeping infants (e.g. Leroy et al., 2011, Anderson et al., 2001) and awake infants (albeit with a high attrition rate; Dehaene-Lambertz et al., 2010). If we are interested in understanding the ontogeny of episodic memory from a neurocognitive perspective, then the benefits of such an approach are obvious, enabling as it would a host of new and exciting questions to be posed. The most basic issue concerns the developmental trajectory of the hippocampus and the various hippocampal subfields. For instance, using high-resolution structural MRI (Bonnici et al., 2012a), we could ask whether or not these distinct regions mature at different rates (as suggested by the monkey work of Lavenex and Banta Lavenex, 2013) and observe and quantify the postnatal structural changes that occur within the hippocampus and related MTL structures. In this way, high-resolution longitudinal data acquired throughout infancy and into childhood could track the developmental trajectory of these structures. Moreover, it would extend the interesting findings of Gogtay et al. (2006) who demonstrated, using structural MRI, that although the volume of the human hippocampus remains remarkably heterogeneous between the ages of 4 and 25 years, significant differences are evident between the posterior (which increases in volume) and the anterior (which decreases in volume) sub-regions over time.

Acquiring structural MRI data around the transitional age of 9 months could also be informative in helping to clarify what exactly occurs at this time, informing the major theoretical accounts of infant memory which speculate that the changes observed in infant memory around this age are driven by hippocampal maturation. However, we could also ask whether such changes are driven by simple time-dependent maturation processes or are triggered by life events such as the onset of independent locomotion (e.g. Herbert et al., 2007).

MRI in infants could, however, potentially provide much more than structural correlates of hippocampal development. For instance, fMRI could enable us to track the development of the episodic memory network. This network is also activated during tasks of prospection, navigation, theory of mind (Buckner and Carroll, 2007), the mental construction of scenes (Hassabis et al., 2007a), and scene viewing (Aly et al., 2013). The overlap between the episodic memory network and the brain areas engaged by these seemingly disparate cognitive processes is unsurprising when the complex nature of episodic memory is considered. For instance, episodic memory depends upon the successful development of a wide range of cognitive functions including associative and relational binding processes, a subjective sense of self in time, and a developed spatial cognition to support the ‘where’ component of episodic memory. Spatial cognition alone (upon which episodic memory is considered to reside; O’Keefe and Nadel, 1978, Burgess et al., 2002, Maguire and Mullally, 2013) represents a complex set of cognitive processes which appear to mature at different rates throughout early and middle childhood (Sluzenski et al., 2004, Sluzenski et al., 2006, Townsend et al., 2010, Bauer et al., 2012, Ribordy et al., 2013, see also Blue et al., 2013, for a discussion of recent data from infant rhesus macaques). Scene viewing, which is a completely passive task in combination with fMRI, could enable us to track the emergence of the neural network supporting the development of the episodic memory, while simultaneously enabling us to establish the functionality of individual components within the episodic memory network, such as the hippocampus, and to assess the cohesiveness of the network as a whole.

The ability to mentally construct scenes is known to depend on the hippocampus in adults (Hassabis et al., 2007b, Mullally et al., 2012). A marker of this is a cognitive phenomenon known as boundary extension, whereby people perceive more of a visual scene than was presented to them. This is a robust, consistent, implicit and automatic cognitive effect that is found in adults (Intraub and Richardson, 1989), children (Seamon et al., 2002) and even in 3- to 7-month-old infants (Quinn and Intraub, 2007). Using an fMRI adapted version of this paradigm (such as in Chadwick et al., 2013) where again participants passively view scenes, the emergence of these scene construction processes, and their relationship with hippocampus and the episodic network, could be explored in infancy. Studies using these kinds of scene viewing paradigms could enable us to infer when adult-like episodic memory may be neurally feasible even if behavioural correlates of episodic memory may not be attainable until linguistic proficiency is achieved.

Clearly, the above paradigms require the cooperation of an awake participant which we acknowledge is not an easy feat in such a young population. One way to circumvent this challenge is to investigate the emergence of the resting-state, as opposed to the episodic memory, network. This is relevant because the episodic memory network shares numerous similarities with the resting-state network – a network of spontaneous and intrinsic brain activity observed in the absence of any overt task performance and during the early stages of sleep (Fukunaga et al., 2006). A number of studies have already begun to explore this network in very young infants. For instance, Gao et al. (2009) found that 2-week-old infants exhibited a primitive and incomplete default network that did not appear to include the hippocampus. By 1-year of age this network showed a marked increase in brain regions demonstrating connectivity but it was not until 2-years of age that this network was comparable to that observed in adults and included the medial prefrontal cortex, the posterior cingulate/retrosplenial cortices, inferior parietal and lateral temporal cortices, and the hippocampus. Thus, a lack of connectivity within the default network would presumably also be evident in the episodic memory network, and would most likely disrupt the long-term consolidation processes necessary to establish enduring episodic memories in regions such as the hippocampus and ventro-medial prefrontal cortex (Bonnici et al., 2012b), and could perhaps explain why very early episodic memories do not appear to be successfully consolidated and accessible in adulthood. Tracking development and time-locking these anatomical and physiological changes to behavioural changes observed in memory development could greatly assist our understanding of the neural substrates of mnemonic processes and potentially enable the distinct contributions of components of this network to be elucidated.

Of note, functional imaging data are also being successfully acquired in awake infants via the use of functional near infrared spectroscopy (fNIRS; Meek et al., 1998). This technique is growing in popularity (Lloyd-Fox et al., 2010, Vanderwert and Nelson, 2013) because it is light, non-invasive and can accommodate a degree of movement which enables an infant to remain seated on their parent's/carer's lap throughout the experiment. However, while fNIRS measures the same haemodynamic response as fMRI, it does not have the spatial resolution of fMRI or the ability to penetrate to structures located deep within the brain. To date, therefore, it is unsuited to studies whose primary goal is to measure the function of the hippocampus and surrounding structures, meaning that such studies must persevere with fMRI and the challenges it poses when attempting to acquire data from a non-sleeping infant. Similar problems are associated with the use of scalp-recorded event-related potentials (ERPs). Although ERPs have been successfully utilised to address important questions about encoding, storage, and consolidation processes in the immature brain (e.g. Bauer et al., 2003, Bauer et al., 2006), the inability of ERPs to penetrate to many of the episodic memory network structures, such as the hippocampus, renders them of limited use when addressing the above theoretical questions.

In addition to studying the neural correlates of infants’ memories, the results of Tustin and Hayne's (2010) study indicate that the earliest memories of young children (<10-years-old) who appear capable of recollecting episodic events from early infancy, could provide important insights into how infants’ very earliest episodic memories are supported at a neural level, and how these differ from episodic memories acquired from later time periods. It is possible that an fMRI analysis technique known as multi-voxel pattern analysis that can be used to ‘decode’ representations of individual episodic memories in the human hippocampus and elsewhere solely from patterns of fMRI activity (Bonnici et al., 2012b, Chadwick et al., 2012), could be particularly useful here. More specifically, it would enable us to track the life of individual episodic memories, hence potentially providing leverage on the phenomenon of infantile amnesia, and allowing ideas such as the neurogenic hypothesis to be tested in the developing human brain. Additionally, the use of fMRI in early childhood, in particular between the ages of 3- and 4-years, where a significant increase in the long-term retention of episodic memories is noted (e.g. Scarf et al., 2013, Morgan and Hayne, 2011) could be helpful in exploring changes in the episodic memory network that may accompany the offset of infantile amnesia. Again, scene-related tasks such as those utilised by Chadwick et al. (2013; see also Mullally et al., 2012, Quinn and Intraub, 2007) could be advantageous as they place no linguistic demands on young participants in whom language abilities are still developing.

In summary, the data we have reviewed above suggest that infants are capable of impressive mnemonic feats. However, these findings raise as many questions as they answer. For instance, does the ability to imitate a sequence of events, or indeed to successfully infer a relationship between two items that have never before been seen together, truly represent the early emergence of episodic memory? If so, are these abilities related to the maturational status of the hippocampus itself, or to the maturation of alternate brain regions (e.g. perirhinal cortex)? Or are they related to a growing cohesion within the episodic memory network (see also Johnson's (2001) interactive specialisation account; cited in Riggins, 2012)? These questions are important as the answers to them will form the cornerstone upon which our understanding of the development of episodic memory (which is believed to persist until the end of the first decade of life; Bauer et al., 2012) will be built. Moreover, addressing the paucity of neuroimaging data in the infant memory literature will also help to deal with the dearth of MRI studies in the wider developmental memory literature (Thomas and Jorgenson, 2012).

## 5 Conclusions

Memory is at the core of our cognitive and social development. Understanding its ontogeny has important implications for childcare and education, and for elucidating how memory is supported in the adult brain. The past few decades have witnessed creative and ingenious ways of examining infants in order to appreciate what they are learning and if they remember, coupled with insights from surface ERP recordings of cortical responses. Models and testing of infant memory have been greatly influenced by a taxonomy that was popularised in the adult literature in the 1980s, which emphasised the dichotomy between declarative or explicit memory, and nondeclarative or implicit memory. Because this taxonomy was in large part driven by data from amnesic patients with hippocampal damage, this scheme has been used to make inferences about the functionality of the infant hippocampus.

However, in recent years the milieu has changed in several respects. First, many of the basic tenets of the memory taxonomy have been challenged leading to a general consensus that distinctions such as explicit/implicit are no longer valid particularly in relation to hippocampal function. Instead, there has been a burgeoning of theories that seek to conceptualise the hippocampal contribution to episodic memory in particular, and indeed to wider cognition, in novel ways that can better account for new data (for more on this see Maguire and Mullally, 2013). Second, findings from the infant memory field, examples of which we reviewed here, undoubtedly indicate that even very young infants have a more adept and flexible memory system than was previously thought. Similarly, recent data also point to the need to finesse our understanding of infantile amnesia. Third, neurobiological evidence from non-humans is starting to accrue that is motivating new hypotheses about hippocampal development, with potential implications for interpreting infant memory data from humans.

Callaghan et al. (2014) recently attempted to renew interest in understanding infantile amnesia, and urged the use of new technologies in molecular biology to unpack the molecular basis of this phenomenon. In a similar vein, here we suggest that it may be time for the infant memory field to take on board new theories of memory and hippocampal function, and embrace technologies such as MRI that could offer a means of progressing points of dispute. To be clear, we are not advocating the abandonment of cognitive testing of infants in favour of fMRI, rather we suggest that the use of MRI could help to motivate and constrain neurocognitive theories of memory development in human infants. Indeed, grounding infant memory in neurobiology may be even more important than for adults given the inability of infants to disclose anything about their own capabilities. The challenges of utilising techniques such as fMRI are substantial, however, the potential rewards we believe could be manifold.

## 6 Conflicts of Interest

All authors declare that there is no conflict of interest.
